# A Novel Physical Fatigue Assessment Method Utilizing Heart Rate Variability and Pulse Arrival Time towards Personalized Feedback with Wearable Sensors

**DOI:** 10.3390/s22041680

**Published:** 2022-02-21

**Authors:** Ardo Allik, Kristjan Pilt, Moonika Viigimäe, Ivo Fridolin, Gert Jervan

**Affiliations:** 1 Department of Health Technologies, Tallinn University of Technology, Ehitajate tee 5, 19086 Tallinn, Estonia; kristjan.pilt@taltech.ee (K.P.); moonika.viigimae@taltech.ee (M.V.); ivo.fridolin@taltech.ee (I.F.); 2 Department of Computer Systems, Tallinn University of Technology, Ehitajate tee 5, 19086 Tallinn, Estonia; gert.jervan@taltech.ee

**Keywords:** physical fatigue, fatigue assessment, heart rate variability, pulse arrival time, wearables

## Abstract

This paper proposes a novel method for physical fatigue assessment that can be applied in wearable systems, by utilizing a set of real-time measurable cardiovascular parameters. Daylength measurements, including a morning test set, physical exercise during the day, and an afternoon test set were conducted on 16 healthy subjects (8 female and 8 male). To analyze cardiovascular parameters for physical fatigue assessment, electrocardiography, pulse wave and blood pressure were measured during the test sets. The fatigue assessment questionnaire score, reaction time, countermovement jump height and hand grip strength were also measured and used as reference parameters. This study demonstrates that (i) the compiled test battery can selectively assess the rested vs. physically-fatigued states; (ii) the obtained linear support-vector machine, trained using the heart rate variability based parameter (F-score 0.842, accuracy 0.813) and pulse arrival time based parameter (F-score 0.875, accuracy 0.875) shows a promising ability to classify between the physically mildly fatigued and significantly fatigued states. Despite the somewhat limited study group size, the results of the study are unique and provide a significant advancement on the existing physical fatigue assessment methods towards a personalized and continuous real-time fatigue monitoring system with wearable sensors.

## 1. Introduction

Fatigue has been used as a term to describe an altered physiological state that results in decreased mental or physical performance, which may be caused by sleep loss, circadian changes or high workload [1,2]. The ability to effectively monitor fatigue is most desired due to multiple reasons since the complaint of fatigue is high in the general population, ranging from 18.3% to 27% [3]. As fatigue can directly influence the mental and physical ability of people to perform even light activities, workers’ fatigue stemming from high demand jobs, long duty periods and accumulative sleep debt is a significant problem in modern industry. The high prevalence of fatigue has likewise been reported in many operational settings to induce safety problems [4]. Whereas previous studies of fatigue have mostly been focused on fatigue tests in different (work) settings, evaluation of muscular fatigue, subjective symptoms of fatigue, indicators of nervous strain, and the practical application of fatigue tests [5], further examination of the prior measures of fatigue addressed in these studies suggests that a practical need for a new multidimensional measure of fatigue exists.

Due to the definitional difficulties and multiple causes of fatigue, no single instrument can be applied as a gold standard for fatigue measurement. Fatigue may also have several confounding factors such as medication, psychological and cognitive conditions, and deconditioning [6,7]. The multi-factorial nature of fatigue suggests that a single universal test to measure fatigue may not exist [8]. Fatigue assessment studies have usually compiled different test batteries of various measures [9,10], which may be classified into six different categories: (i) questionnaires on subjective feelings of fatigue, (ii) psychological tests, (iii) neuropsychological tests, (iv) biochemical indexes, (v) physiological tests and (vi) autonomic nervous function tests [8].

It is important to note that most of these measurement methods require special conditions and testing environment and are therefore not suitable for real-time assessment of physical fatigue. Yet, human activity monitoring has advanced towards utilizing new wearable technologies and devices, which are able to conveniently measure, collect and analyze the user’s physiological data [11]. Although a considerable effort is being made in wearable sensors, for comfortable use, wearables would need to be small and unobtrusive, which in turn requires keeping the power consumption and computational power as low as possible [11,12]. For these reasons, in order to assess fatigue continuously or repeatedly by wearable sensors, a novel approach that utilizes real-time measurable physiological signals will be needed.

This study proposes a set of cardiovascular (CV) parameters for physical fatigue assessment, which could be measured continuously in real-time. Since only heart rate variability (HRV) and reaction time (RT) were explored in an earlier study on physical fatigue [13], it can be hypothesized that compiling an enhanced test-battery of real-time CV parameters could yield a more effective outcome regarding the reference parameters for physical fatigue assessment. Correlations in-between the test battery parameters were analyzed on the individual level, which to the best of the authors’ knowledge, has not been performed in the previous physical fatigue studies. A strong correlation between various measures could be used to improve the overall assessment quality or decrease the required computational power and complexity of the measurement system by removing redundant parameters. Building a model based on the data for classifying between different fatigue states would provide a basis for further development towards a continuous real-time fatigue monitoring system.

This study introduces a method for real-time physical fatigue assessment that can be applied in wearable systems, by utilizing a set of real-time and easily measurable cardiovascular (CV) parameters.

## 2. Materials and Methods

### 2.1. Study Design

**Figure 1:**
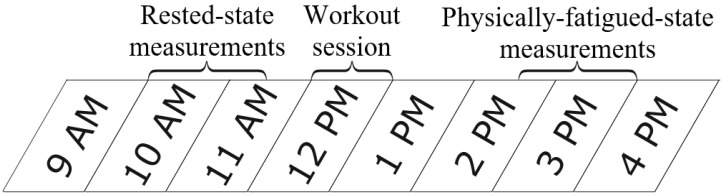
Overview of an experiment day. Cardiovascular and reference parameters were measured similarly in both test sets. The workout session involved multiple full-body exercises.

An experiment was conducted on all the subjects (*n* = 16) in three main activities, including measurements in the morning and afternoon with a workout session in-between (Figure 1). The calculated or measured reference parameters included the fatigue assessment questionnaire score, reaction time (RT), hand grip strength and countermovement jump (CMJ) height. The evaluated CV parameters included heart rate (HR), measures of heart rate variability (HRV) and blood pressure normalized pulse arrival time (PAT).

Rested-state (RS) measurements were obtained in the morning during 10:00–11:30. Following the RS measurements, all subjects performed the same exhausting full-body workout to induce physical fatigue. The 60-min workout was comprised of multiple sets of various exercises such as squats, burpees, sit-ups, push-ups, planks and jumping jacks. Physically-fatigued-state (PFS) measurements were obtained in the afternoon during 14:30–16:00. All measurements were conducted by the same person with the aim to avoid interoperator variability.

### 2.2. Study Group

The study group was comprised of 16 healthy 18 to 48 year-old subjects (8 female and 8 male) with the anthropometric parameters outlined in Table 1. The subjects signed a consent form prior to being enrolled in the experiment and the procedures performed were in accordance with the established ethical standards. The experiment was approved by the Tallinn Medical Research Ethics Committee (No. 1954).

To reduce the effects of possible confounding factors, the following individuals were excluded from the study group: (i) individuals suffering from a disease or condition causing significant fatigue (congestive heart failure, respiratory failure, cancer, anemia, sleep disorders or a major psychiatric condition); (ii) individuals taking medicines that cause fatigue (beta blockers, diuretics or narcotics); (iii) pregnant or breastfeeding women; (iv) individuals that had worked night shifts within the past month; (v) individuals that consume alcoholic drinks daily, use of illicit drugs or smoking; (vi) individuals that had experienced a serious illness within two weeks before the test. The subjects of the study were asked to avoid any hard workout 24 h before the test and not to consume caffeinated drinks or foods.

### 2.3. Test Battery Design

Most of the parameters were chosen based on the literature overview of previous fatigue assessment studies. The analyzed parameters were divided into reference parameters that usually need administered tests and cannot be obtained in real-time, and CV parameters that could be continuously monitored and measured. These parameters were measured in the same order in both the rested and physically-fatigued states. The selected reference parameters included the score of a fatigue questionnaire [9], RT [10,14,15], hand grip strength [10,16] and CMJ height [9,10,16]. The evaluated CV parameters involved HR [1,17], HRV [1,2,18] and PAT. The parameters were selected with the aim to keep the complexity of the overall measurement process and computational power requirements as low as possible for suitable use in wearable systems, and thus, only time-domain measures were considered. While the effects of muscle fatigue on HRV have been additionally analyzed using frequency-domain and non-linear measures [19], these were not explored due to the following reasons: (i) information captured by frequency-domain parameters has been found to correlate with the measures analyzed in this study [20]; (ii) transforms needed for spectral analysis require extra resources.

### 2.4. Fatigue Questionnaire

At the start of the experiment the subjects were asked to complete a questionnaire to evaluate their current subjective fatigue level. The questionnaire adopted for the experiment was the Swedish Occupational Fatigue Inventory (SOFI), developed for the measurement of after-work fatigue [21] (Figure S1). The scale items were scored based on a 7-point Likert scale to assess fatigue from 0 (not at all) to 6 (to a very high degree). The scale items were as follows: (i) physical exertion (having palpitations, sweaty, out of breath and breathing heavily); (ii) physical discomfort (tense muscles, numbness, stiff joints and aching); (iii) lack of motivation (lacking concern, passive, indifferent and uninterested); (iv) sleepiness (falling asleep, drowsy, yawning and sleepy); (v) lack of energy (worn out, spent, drained and overworked). The score of the questionnaire was analyzed as the percentage of the maximum score.

### 2.5. Reaction Time Measurement

Subject RT was measured using the PC-PVT platform developed and validated for psychomotor vigilance testing [22,23]. The test was conducted on a desktop computer (CPU: Intel Core i5-7500, GPU: Intel HD Graphics 630 (Intel, Santa Clara, CA, USA)), Mouse: Logitech G203 (Logitech, Lausanne, Switzerland)) with an external monitor (HP E233, Hewlett-Packard, Palo Alto, CA, USA). The protocol was selected to be similar to the one applied in previous fatigue assessment studies [10]. During a 5-min test each subject performed about 75 simple RT measurements. Similar to multiple other RT measurement studies, in this study, the inter-stimulus interval was selected between 3 to 5 s [14].

### 2.6. Hand Grip Strength Measurement

Hand grip strength was measured using Grip Force Transducer dynamometer (MLT004/ST, ADInstruments, Sydney, Australia) with PowerLab 4/25T (ADInstruments, Sydney, Australia) data acquisition device and LabChart software (v. 8.1.13, ADInstruments). The subjects performed five maximal voluntary contractions with the dominant arm while seated. Hand grip strength was analyzed as the average of the maximums of the five repetitions.

### 2.7. Countermovement Jump Measurement

**Figure 2:**
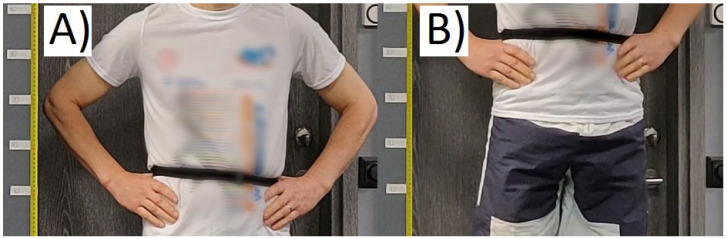
Countermovement jump (CMJ) height measurements. The subject is (**A**) standing and (**B**) at the maximum height. CMJ height was found as the vertical difference of the belt, which was firmly attached around the torso.

Each subject performed five maximal effort CMJ according to the recommended method [24]. The subjects were instructed to have their feet shoulder-width apart and hold their hands on their hips while performing the test. The performance was filmed and recorded at 60 frames per second with a camera (OnePlus 6, OnePlus Technology, Shenzhen, China), which was statically mounted at a fixed distance from the subject. Based on the recording, the height of each jump was calculated as the difference between the distance of a marked position (below the ribcage of the subject’s torso) from the ground while standing and at the maximum jump height (Figure 2). The performance was estimated as the average of the jump heights of each CMJ repetition.

### 2.8. Veloergometer Test

Following CMJ measurements, a veloergometer test was performed on the Tunturi T6 Alpha 300 veloergometer (Tunturi, Turku, Finland). During the test, signals were measured to calculate different parameters based on heart electrical activity and pulse waveform in the resting and recovery phases between cycling. The test schedule is shown in Table 2. The subject sat on the veloergometer saddle calmly for at least 3 min prior to the heart rate measurement in a 5-min resting phase. Then the subject cycled at 60 rotations per minute on three different power levels (60 W, 90 W and 120 W) for three minutes with each level test followed by a 5-min recovery phase [18,25,26]. The power levels were manually changed during the recovery phases by the test supervisor.

### 2.9. Heart Electrical Activity Measurement

**Figure 3:**
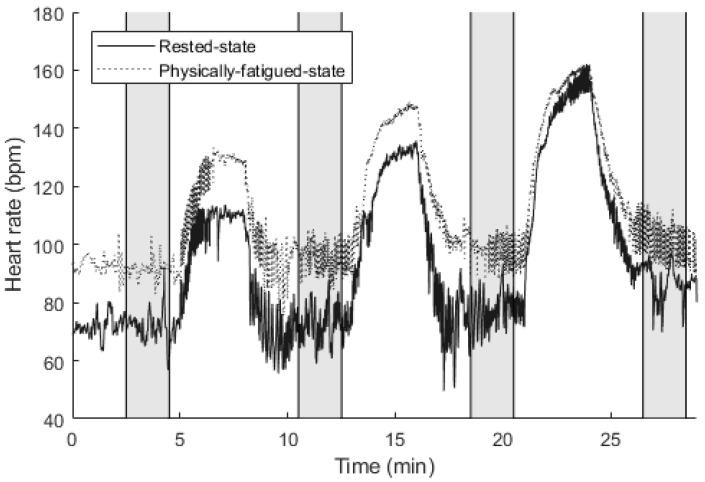
Heart rate of a subject during the veloergometer test in the rested state and the physically-fatigued state. The heart electrical activity parameters were calculated based on the grayed areas.

Subject ECG signals were recorded during the veloergometer test at the sampling rate of 1 kHz using the PowerLab 4/25T (ADInstruments, Sydney, Australia) data acquisition device and LabChart software (v. 8.1.13, ADInstruments). HR and HRV parameters were calculated based on the measured ECG signals to compare the heart electrical activity between the rested state in the morning and the physically-fatigued state in the afternoon. R-peaks of the ECG signals were detected by adopting the Hamilton–Tompkins algorithm [27], and manually verified in order to eliminate any errors. An example of subject HR in the veloergometer test is displayed in Figure 3.

HRV parameters assessed in the test include time-domain measures SDNN (standard deviation of all NN intervals) and RMSSD (square root of the root mean square of the sum of all differences between successive NN intervals), which are the two commonly employed HRV parameters for the analysis of heart electrical activity [28]. The values of heart electrical activity parameters were calculated based on a 2-min signal section during the ‘slow phase’ of heart rate recovery [18], which was selected between 2.5 and 4.5 min during the resting phase and after cycling (grayed areas in Figure 3).

### 2.10. Pulse Arrival Time Measurement

**Figure 4:**
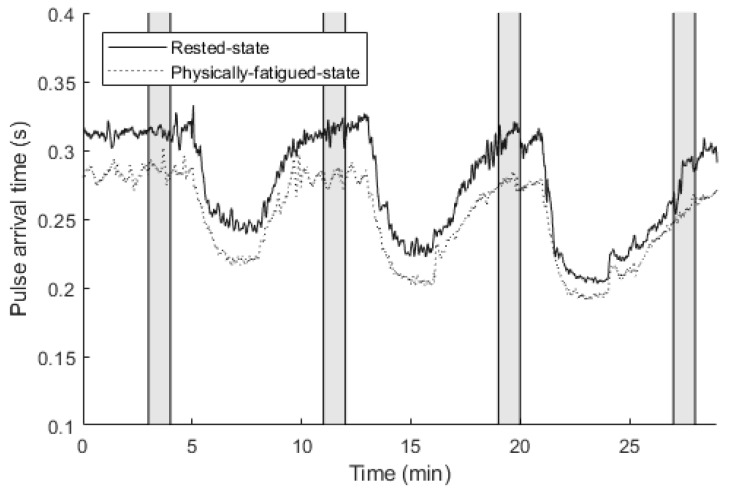
Pulse arrival time of a subject (before normalizing with blood pressure) during veloergometer test in the rested state and the physically fatigued state. Parameter values were calculated based on the grayed areas.

While prior studies have explored PAT in exercise settings [29], this study is likely to be the first one to evaluate PAT for physical fatigue assessment. The pulse wave was registered synchronously with the heart electrical activity parameters employing the same sensing unit with an external piezoelectric transducer attached to the fingertip (MLT 1010 pulse transducer, ADInstruments, Sydney, Australia). PAT was found as the time difference between the ECG R-peak and the pulse wave signal rising front. PAT values were calculated based on a 1-min-signal section, selected between 3 and 4 min during the resting phase and after cycling. These sections and PAT of a sample subject are presented in Figure 4.

With the aim to reduce intersubject variability, PAT values were subsequently normalized based on blood pressure measurements, conducted in the same time sections [30]. The values were normalized to the systolic blood pressure of 120 mmHg, adopting the relation of every 1 mmHg difference causing 1 ms discrepancy in PAT [31].

### 2.11. Statistical Analysis

The test battery parameters were presented as mean +/− standard deviation (SD) over all subjects for the rested state and the physically-fatigued state. The paired *t*-test (*p* < 0.05) was used to find statistical difference between the measurements.

For every test battery parameter, the percentual change between the rested state and the physically-fatigued state was found individually for each subject. A linear correlation coefficient was calculated separately for each parameter pair to detect any linear relationship.

### 2.12. Grouping Based on Fatigue Levels

While all the subjects followed the same study protocol, they experienced different levels of physical fatigue based on their physiological and physical background. To distinguish between the mildly fatigued and significantly fatigued, the subjects were arranged into two groups in regard to their relative change in CMJ height between the rested state and the physically-fatigued state. From the measured reference parameters, CMJ height was selected since (i) two reference parameters, CMJ height and questionnaire score, had the ability to differentiate the rested state and the physically-fatigued state; (ii) compared to questionnaire score, CMJ height was seen as a more objective parameter.

All CV parameters (and multiple subparameters) were analyzed individually to test selectivity against the mildly fatigued and significantly fatigued groups. The total number of parameters analyzed was 74, including the values of SDNN, RMSSD, PAT and HR of different veloergometer test phases and subject fatigue states.

The parameters were evaluated by creating a decision stump using MATLAB’s function *fitctree* that returns a fitted binary classification decision tree based on the input parameter. The leave-one-out cross-validation scheme was employed, where for each subject the decision stump was created based on the values measured from all other subjects. Applying this method, F-score and accuracy were found for the evaluation of each parameter.

Finally, a linear support vector machine (SVM) model was trained based on the two best performing parameters for binary classification between the mildly fatigued and the significantly fatigued groups. This classifier has been given as a valid example of a possible use of the findings of this study. The model was trained based on all the participants using MATLAB’s function *fitcsvm* and the decision boundary was given using the formula:

w_1_x_1_ + w_2_x_2_ + b = 1, (1)

where w_1_ and w_2_ are the coefficients for the parameters x_1_ and x_2_ and b is the bias term.

## 3. Results

### 3.1. Average Parameter Values

From the measured reference parameters, the questionnaire score had an increase of 15.2% for the whole study group, 17.9% for the female subgroup and 12.5% for the male subgroup for the rested vs. physically-fatigued states (see Table 3). This should confirm that the subjects felt more tired during the physically-fatigued state as compared to the rested state. CMJ height had a statistically significant decrease for the whole group −3.1% and for the female subgroup −2.7%, but interestingly not for the male subgroup (−1.9%). While the average RT increased by 1.1% and hand grip strength decreased −2.9% for the whole group, these changes were not found statistically significant (*p* = 0.458; *p* = 0.113).

For CV parameters (as in Table 4), only HR had a statistically significant increase for all study groups, including 9.5% for the whole group, 9.6% for the male subgroup and 9.4% for the female subgroup. Heart variability parameters SDNN (−21.2%, −19.6% and −23.2%, respectively) and RMSSD (−29.3%, −32.0% and −25.9%, respectively) had a statistically significant decrease for the whole group and the female subgroup, but not for the male subgroup (*p* = 0.102). The changes in average values of blood pressure normalized PAT (−2.0%, −4.7% and 0.7%) were not statistically significant (*p* = 0.172).

### 3.2. Correlation

Relatively strong linear correlation (0.5 < R < −0.5) was noted between several test battery measures (Table 5). For the whole group, these levels were found between HRV parameters SDNN and RMSSD (0.71); HR and SDNN (−0.61); HR and RMSSD (−0.57). For the male subgroup, the same levels were found between the questionnaire score and hand grip strength (0.74), CMJ height (−0.59); hand grip strength and CMJ height (−0.59), PAT (−0.80), HR (−0.53); SDNN and RMSSD (0.93), HR (−0.88); between RMSSD and HR (−0.80). For the female subgroup, linear correlation levels were found between questionnaire score and hand grip strength (−0.55); RT and hand grip strength (−0.80), SDNN (0.56), HR (−0.63); hand grip strength and SDNN (−0.74); PAT and HR (−0.79).

### 3.3. Grouping Subjects Based on Fatigue States

**Figure 5:**
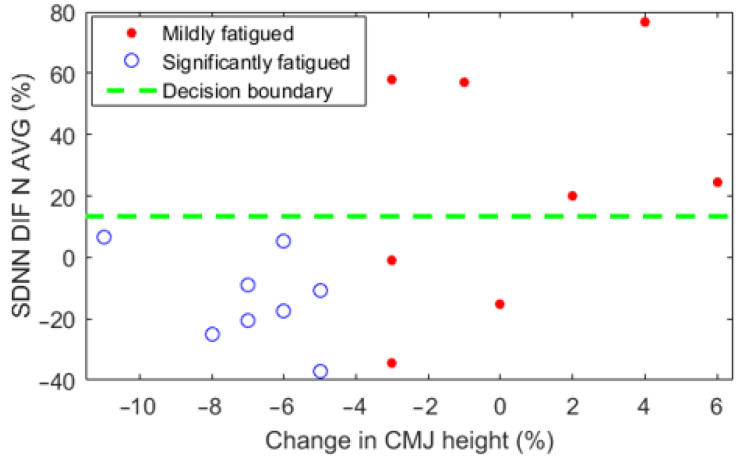
Change in CMJ height value compared to SDNN_DIF_N_AVG value. The subjects were arranged into the mildly fatigued and significantly fatigued groups. The shown threshold is found based on the data from all the subjects.

**Figure 6:**
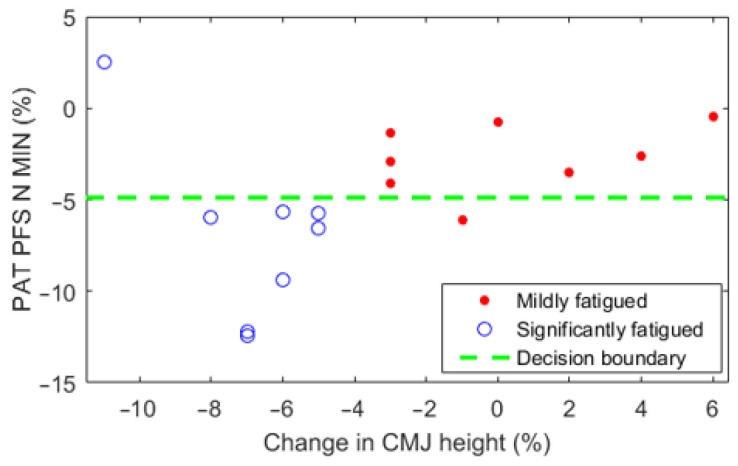
Change in CMJ height value compared to PAT_PFS_N_MIN value. The subjects were arranged into the mildly fatigued and significantly fatigued groups. The shown threshold is found based on the data from all the subjects.

In order to test how effectively the real-time measurable parameters differentiate between the mildly fatigued and significantly fatigued study groups, all these parameters (and multiple subparameters) were analyzed individually by conducting a classification task using only the chosen feature. Based on these findings, the two highest performing parameters were found as follows:

1. Relative change of the resting SDNN value normalized with the average recovery phase value between the rested-state and the physically-fatigued-state SDNN_DIF_N_AVG (F-score 0.842, accuracy 0.813) (Figure 5).
2. Resting PAT value normalized with the lowest recovery phase value during the physically-fatigued-state PAT_PFS_N_MIN (F-score 0.875, accuracy 0.875) (Figure 6).

**Figure 7:**
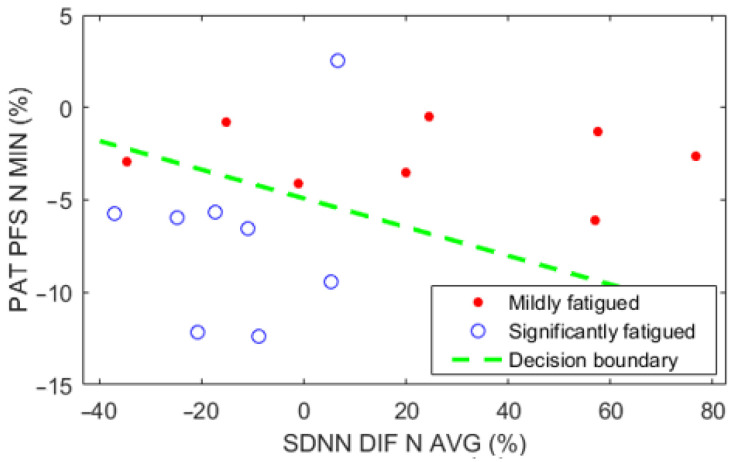
Linear SVM model for binary classification between the mildly fatigued and significantly fatigued groups.

These two parameters were used to train a linear SVM to classify new subjects or users into the mildly fatigued group or the significantly fatigued group (Figure 7). The linear SVM obtained demonstrates a promising ability to classify between the mildly fatigued or significantly fatigued physical states. The decision boundary can be described with the following formula according to (1):

−0.0261x_1_ − 0.3366x_2_ − 1.6558 = 0 (2)

where x_1_ is the parameter SDNN_DIF_N_AVG and x_2_ is the parameter PAT_PFS_N_MIN.

## 4. Discussion

This study has evaluated how exercise induced physical fatigue affects various test battery measures, and whether real-time measurable cardiovascular (CV) parameters could provide sufficient data to classify between the mildly fatigued and significantly fatigued groups with the aim to provide information for real-time physical fatigue assessment. The main findings can be listed as follows: (i) from the assessed cardiovascular parameters, the statistically significant change between the rested state and physically-fatigued state was noted in the average heart rate and heart rate variability measures SDNN and RMSSD; (ii) the strongest linear correlation was found between the reference parameter hand grip strength and CV parameter pulse arrival time (PAT); (iii) the finest CV parameters for separating the mildly fatigued and significantly fatigued groups were based on heart rate variability (HRV) parameter SDNN between the rested state and the physically-fatigued state and PAT changes during the physically-fatigued state.

While most study parameters were selected drawing on the findings of previous studies, not all of the parameters were found significant based on the results of this study. From the reference parameters, the score of fatigue questionnaire showed a statistically significant increase (of about 15.2%) between the rested-state and the physically-fatigue-state data, which is consistent with the previous findings [3]. Countermovement jump (CMJ) height decrease was statistically different for the whole study group (average decrease of 3.1%) and the female subgroup, remaining in the same range as found in the previous studies [3,10,16]. In accordance with the previous studies, the average value of reaction time (RT) increased (1.3%) [10,14,15] and hand grip strength decreased (−2.9%) [10,16]; however, these changes were not found statistically significant. The results of the hand grip strength test could be explained by the full-body workout regime that did not involve a sufficient number of exercises for the specific arm muscles. It was expected that RT would decrease due to physical fatigue [13]; however, the present study did not reach such a result. A possible explanation for the difference in the data obtained in previous studies may be that different modes of physical work or exercise were used. It is also likely that the fatigue level during physically-fatigued-state measurements was not sufficiently high to limit the inhibitory control of some subjects [32].

From the evaluated CV parameters, the average heart rate had a statistically significant increase of 9.5%, which is in accordance with the previous studies [1,17]. The HRV parameters SDNN and RMSSD decreased by 21.2% and 29.3%, respectively, between the rested-state and the physically-fatigued-state measurements, which has also been noted by other studies [1,2,18]. The presence of some outliers in the results of the effects of fatigue on the cardiovascular parameters may be due to a faster and better recovery of these subjects compared to others [33]. It is interesting to note that PAT, which is a novel parameter for physical fatigue assessment studies, had a decrease of 2.0% for the whole group and 4.7% for the female subgroup, but for the male subgroup the value increased by 0.7%.

The linear correlation coefficient was found based on the relative individual changes between all measures. The strongest correlation between CV and reference parameter for the whole group was found between hand grip strength and PAT (linear correlation coefficient of −0.39). This finding was consistent with the date obtained in the male subgroup, where the linear correlation coefficient was −0.80; however, for the female subgroup the strongest correlation was found between HRV measure SDNN and hand grip strength (−0.79).

In total, 74 different subparameters were evaluated based on how effectively they classify between the mildly fatigued and significantly fatigued study groups compiled using the relative change in CMJ height value. These parameters were found using SDNN, RMSSD, PAT and HR values from different veloergometer test phases and subject fatigue states. The finest parameters for differentiating between these groups were the relative change of the resting SDNN value normalized with average recovery phase value between the rested-state and physically-fatigued-state measurements (SDNN_DIF_N_AVG; F-score 0.842, accuracy 0.813) and the resting PAT value normalized with the lowest recovery phase value during the physically-fatigued-state (PAT_PFS_N_MIN; F-score 0.875, accuracy 0.875). A simple linear support vector machine model was trained based on these two parameters to provide a valid example of a possible application of these results. This model, when implemented into a real-time monitoring system, has a potential to reveal whether the user is ‘mildly fatigued’ or ‘significantly fatigued’ after a physically demanding day.

Based on the findings of this study, it can be concluded that the test battery devised for the experiment has an added value for the assessment of physical fatigue. The evaluated CV parameters showed promising results compared to the reference parameters and thus could be used for real-time physical fatigue monitoring in workplace settings and for the general population. Novel parameters based on PAT were also found to provide additional information for the improvement of the overall quality of physical fatigue assessment.

This study has encountered several limitations that should be considered in the following studies, namely: (i) as the study was conducted on a relatively small number of subjects, the results should be verified on a larger study group; (ii) since the induced physical fatigue is likely to have been specific to the used workout and study protocol, it should be further explored how different workout regimes affect the results; (iii) the experiments were conducted in the lab settings and should thus be verified in real-life situations; (iv) it is possible that extra information for fatigue assessment could be obtained by additional exploration into frequency-domain parameters. While the above limitations should not reduce the impact of this novel study, further research is required to fully evaluate the effectiveness of the test battery compiled for the study in order to determine the sensitivity of variables with the accumulation of fatigue.

## 5. Conclusions

This study has proposed a novel method for real-time physical fatigue assessment employing a set of real-time and easily measurable cardiovascular (CV) parameters. Induced physical fatigue was found to cause statistically significant change in the score of the fatigue questionnaire and countermovement jump height. For the cardiovascular parameters assessed, the statistically significant change was noted in the average heart rate and heart rate variability measures SDNN and RMSSD. The subparameters based on heart rate variability and blood pressure normalized pulse wave arrival time showed the highest performance in classifying between the mildly fatigued and the significantly fatigued groups. The findings of the study can be used to enhance the existing physical fatigue assessment methods and provide a solid ground for further research in the development of a continuous real-time fatigue monitoring system. Further studies with a larger study group will be required to verify the obtained findings in multiple real-life situations.

## Figures and Tables

**Table 1:** Subject anthropometric parameters. Age (years), height (cm), weight (kg) and body mass index (BMI) (kg/m2) mean values with standard deviations (SD).

Count	Age Mean ± SD; Range	Height Mean ± SD; Range	Weight Mean ± SD; Range	BMI Mean ± SD; Range
Total (16)	28.3 ± 7.9; 18–48	173.9 ± 8.1; 163–190	69.9 ± 12.3; 55–91	23.0 ± 2.9; 18.3–30.1
Female (8)	28.4 ± 7.0; 18–42	169.1 ± 5.9; 163–180	63.9 ± 10.5; 55–89	22.4 ± 3.5; 18.3–30.1
Male (8)	28.3 ± 9.2; 18–48	178.6 ± 7.3; 166–190	75.9 ± 11.4; 60–91	23.7 ± 2.2; 20.4–26.4

**Table 2:** Schedule of the veloergometer test.

Activity	Duration in Minutes
Resting	5
Cycling @ 60 W	3
Recovery	5
Cycling @ 90 W	3
Recovery	5
Cycling @ 120 W	3
Recovery	5

**Table 3:** Reference parameter values. Average (mean ± SD) values for the reference parameters in the rested state (RS), physically-fatigued state (PFS) and their difference in percentage (DIF). Results are shown for the whole study group (A), female subgroup (F) and male subgroup (M). Q—questionnaire, RT—reaction time, DYN—dynamometer hand grip force and CMJ—countermovement jump height. Values marked with asterisk (*) indicate statistical difference (paired *t*-test, *p* < 0.05).

		Q (%)	RT (ms)	DYN (N)	CMJ (cm)
RS	A	14.0 ± 7.6	208.7 ± 11.3	360.3 ± 99.1	38.2 ± 8.7
	F	12.1 ± 9.4	206.8 ± 13.4	294.2 ± 47.4	33.1 ± 3.3
	M	15.8 ± 5.3	210.6 ± 9.4	426.4 ± 93.8	43.3 ± 9.7
PFS	A	29.2 ± 13.0	211.4 ± 16.9	349.7 ± 105.7	37.0 ± 9.0
	F	30.0 ± 17.7	211.7 ± 17.2	286.4 ± 48.7	31.6 ± 3.3
	M	28.3 ± 6.9	211.0 ± 17.9	413.0 ± 111.3	42.5 ± 9.8
DIF (%)	A	15.2% *	1.3%	−2.9%	−3.1% *
	F	17.9% *	2.4%	−2.7%	−4.5% *
	M	12.5% *	0.2%	−3.1%	−1.9%

**Table 4:** Cardiovascular parameter values. Average (mean ± SD) values for the cardiovascular parameters in the rested state (RS), physically-fatigued state (PFS) and their difference (value, %) (DIF). Results are shown for the whole study group (A), female subgroup (F) and male subgroup (M). HR—average heart rate, SDNN—HRV parameter SDNN value, RMSSD—HRV parameter RMSSD and PAT—pulse arrival time. Values marked with asterisk (*) indicate statistical difference (paired *t*-test, *p* < 0.05).

		HR (bpm)	SDNN (ms)	RMSSD (ms)	PAT (ms)
RS	A	98.5 ± 10.9	58.0 ± 19.7	35.4 ± 18.9	273.4 ± 21.6
	F	100.6 ± 9.7	52.8 ± 13.3	31.7 ± 12.7	267.5 ± 15.7
	M	96.4 ± 12.3	63.2 ± 24.3	39.0 ± 24.0	279.4 ± 25.9
PFS	A	107.9 ± 12.2	45.7 ± 15.9	25.0 ± 13.8	268.1 ± 23.8
	F	110.1 ± 11.4	40.5 ± 16.0	23.5 ± 17.6	254.8 ± 11.8
	M	105.6 ± 13.4	50.8 ± 15.1	26.5 ± 9.5	281.3 ± 26.0
DIF (%)	A	9.5% *	−21.2% *	−29.3% *	−2.0%
	F	9.4% *	−23.2% *	−25.9% *	−4.7%
	M	9.6% *	−19.6%	−32.0%	0.7%

**Table 5:** Linear correlation coefficient values between different parameters based on the whole study group (A), male subgroup (M) and female subgroup (F). Parameter values are taken as the difference in % between those in the rested state and physically-fatigued state. Q—questionnaire, RT—reaction time, DYN—dynamometer hand grip force, CMJ—countermovement jump height, SDNN—HRV parameter SDNN, RMSSD—HRV parameter RMSSD, PAT—pulse arrival time and HR—average heart rate (between the resting heart rate and average veloergometer cycling heart rate). Values above 0.5 or below −0.5 are marked in bold.

		RT	DYN	CMJ	SDNN	RMSSD	PAT	HR
Q	A	0.36	−0.18	**−0.43**	0.13	0.02	−0.05	−0.13
	F	0.36	−0.55	−0.35	0.10	−0.07	0.18	0.02
	M	0.36	0.74	−0.59	0.36	0.26	−0.36	−0.49
RT	A		−0.24	−0.25	0.11	−0.03	0.03	−0.33
	F		−0.8	−0.04	0.56	0.03	0.41	−0.63
	M		0.40	−0.38	−0.13	0.01	−0.22	−0.04
DYN	A			**−0.10**	−0.24	0.11	**−0.39**	**−0.10**
	F			0.30	−0.74	0.19	−0.18	0.22
	M			−0.59	0.23	0.12	−0.80	−0.53
CMJ	A				−0.09	0.03	0.35	−0.09
	F				−0.29	0.23	0.26	−0.35
	M				−0.08	−0.18	0.25	0.18
SDNN	A					**0.71**	0.30	**−0.61**
	F					**0.10**	0.24	**−0.33**
	M					**0.93**	0.23	**−0.88**
RMSSD	A						0.24	**−0.57**
	F						−0.12	**−0.31**
	M						0.42	**−0.80**
PAT	A							−0.35
	F							−0.79
	M							0.13

## Data Availability

The data presented in this study are available on request from the corresponding author.

## Supplementary Materials

The following materials are available online at https://www.mdpi.com/1424-8220/22/4/1680/s1, Figure S1: The Swedish Occupational Fatigue Inventory questionnaire [21], used to evaluate current subjective fatigue levels of the subjects.
