# Supplementary material for: A Novel Physical Fatigue Assessment Method Utilizing Heart Rate Variability and Pulse Arrival Time towards Personalized Feedback with Wearable Sensors
Source: Sensors (Basel). 2022 Feb 21;22(4):1680. doi: 10.3390/s22041680 (PMC8924887; doi:10.3390/s22041680)
Supplement: Supplementary file 1 [file sensors-22-01680-s001.zip › sensors-1526510-supplementary.pdf]

		nothing to maximal						
1.	Physical exertion<br>(palpitations, sweaty, out of breath, breathing heavily)	0	1	2	3	4	5	6
2.	Physical discomfort<br>(tense muscles, numbness, stiff join, aching)	0	1	2	3	4	5	6
3.	Lack of motivation<br>(lack of concern, passive, indifferent, uninterested)	0	1	2	3	4	5	6
4.	Sleepiness<br>(falling asleep, drowsy, yawning, sleepy)	0	1	2	3	4	5	6
5.	Lack of energy<br>(worn out, spent, drained, overworked)	0	1	2	3	4	5	6
		Total Score:						

Figure S1. The Swedish Occupational Fatigue Inventory questionnaire [19], which was used to evaluate subjective current fatigue levels of the participants.
